# Bcl-2 family proteins in breast development and cancer: could Mcl-1 targeting overcome therapeutic resistance?

**DOI:** 10.18632/oncotarget.2792

**Published:** 2015-02-12

**Authors:** Michelle M. Williams, Rebecca S. Cook

**Affiliations:** ^1^ Department of Cancer Biology, Vanderbilt University School of Medicine, Nashville TN 27232, USA; ^2^ Department of Vanderbilt Ingram Cancer Center, Nashville, TN 37232, USA

## Abstract

Apoptosis, cell death executed by caspases, is essential to normal breast development and homeostasis. Pro-apoptotic and anti-apoptotic signals are tightly regulated in normal breast epithelial cells. Dysregulation of this balance is required for breast tumorigenesis and increases acquired resistance to treatments, including molecularly targeted therapies, radiation and chemotherapies. The pro-apoptotic or anti-apoptotic Bcl-2 family members interact with each other to maintain mitochondrial integrity and regulate cellular commitment to apoptosis. Among the anti-apoptotic Bcl-2 family members, Mcl-1 is uniquely regulated by numerous oncogenic signaling pathways. This review will focus on the role of Bcl-2 family proteins in normal breast development, breast tumorigenesis and acquired resistance to breast cancer treatment strategies, while highlighting Mcl-1 as a promising target to improve breast cancer tumor cell killing.

## INTRODUCTION

The mammary gland is a dynamic tissue that undergoes many developmental stages, including puberty, pregnancy, lactation, post-lactational involution and lobular (menopausal) involution. Normal progression through these stages is regulated by numerous intracellular signaling cues that regulate the balance of mammary epithelial cell (MEC) proliferation and cell death. During puberty and pregnancy, MEC proliferation in the ductal and alveolar MEC lineages, respectively, outweighs cell death, allowing for expansion of each MEC population as needed. In contrast, cell death predominates over proliferation to cull milk-producing MECs once offspring are weaned, and to support lobular involution at menopause. In the breast, as in many other tissues, the cellular commitment to apoptosis is regulated by the intrinsic apoptotic pathway, which is comprised of the Bcl-2 family of proteins.

Two main subclasses of Bcl-2 proteins exist: anti-apoptotic (Bcl2-A1, Bcl-2, Bcl-xL, Bcl-w and Mcl-1) and pro-apoptotic (Bad, Bak, Bax, Bid, Bik, Bim, Hrk, Noxa and Puma). Complex interactions among pro- and anti-apoptotic family members integrate signaling information to regulate cell death decisions. Due to the dynamic nature of the breast, characterized by sequential cycles of cell growth and cell death, Bcl-2 proteins are essential for development and homeostasis. Dysregulation of Bcl-2 proteins can impede development at several key stages. Furthermore, sustained Bcl-2 family dysregulation contributes to evasion of cell death, a hallmark of cancer. In the context of breast cancers, Bcl-2 dysregulation promotes innate or acquired treatment resistance. This review will focus on the role of Bcl-2 family members in breast development, tumorigenesis and therapeutic resistance, and will highlight one member, Mcl-1, as a promising therapeutic target in breast cancers.

### Bcl-2 proteins

The intrinsic apoptotic pathway (IAP) is characterized by mitochondria outer membrane permeabilization (MOMP), resulting in release of cytochrome-c into the cytoplasm. This apoptotic pathway is governed by hierarchical interactions between pro-apoptotic and anti-apoptotic Bcl-2 proteins. Every Bcl-2 family member contains at least one of the four Bcl-2 homology (BH) domains that are highly conserved from *Caenorhabditis elegans* to humans. BH3 domains facilitate and stabilize protein-protein interactions within the Bcl-2 family, which are critical for regulating MOMP [1].

**Figure 1:**
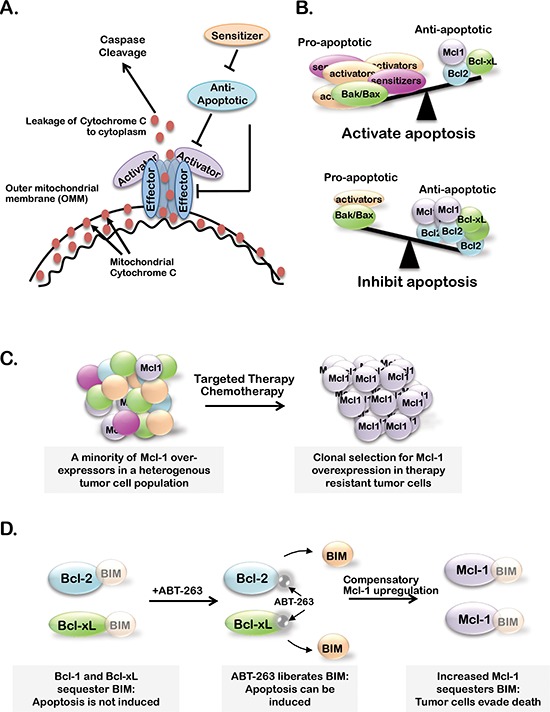
**(A)** Bcl-2 family member effector proteins (Bax and Bak) oligomerize and permeablize the outer mitochondrial membrane (OMM) upon activator-protein (Bid, Bim and Puma) bindingCytochrome-c is able to escape the mitochondria through Bak/Bax pores, leading to caspase cleavage and apoptosis. Anti-apoptotic Bcl-2 family members (A1, Bcl-2, Bcl-xL, Bcl-w and Mcl-1) inhibit apoptosis by preventing effector protein oligomerization or Bak/Bax activation. Sensitizer proteins (Bad, Bik and Noxa) counteract anti-apoptotic members by sequestering anti-apoptotic proteins. **(B)** When the balance of active Bcl-2 proteins favors pro-apoptotic over anti-apoptotic family members, apoptosis is initiated. **(C–D)** Proposed models of Mcl-1 expression or activity supporting resistance to standard chemotherapies or targeted therapies (C) and BH3-mimetics (B).

Although not fully understood, most proposed mechanisms of MOMP suggest that Bcl-2 family effectors (Bak and Bax) heterodimerize to form higher-order oligomers, creating pores in the outer mitochondrial membrane (OMM) permitting cytochrome-c to escape into the cytoplasm [2, 3] (Figure 1A). Thus, activity of Bak/Bax is highly regulated at many levels. One level of regulation contributing to Bak/Bax interaction is subcellular localization. Bak is associated with the OMM and sustains base-line activity of the intrinsic apoptotic pathway [4]. Meanwhile, Bax shuttles from the cytoplasm to the mitochondria in response to apoptotic cues, suggesting that Bax localization adjusts the sensitivity of cells to apoptotic stimuli. A cell with high Bak/Bax expression at the OMM is more ‘primed’ for cell death through the intrinsic apoptotic pathway. Additionally, Bak/Bax harbor BH1-3 domains required for Bak/Bax to interact with each other. However, Bak/Bax cannot interact until they undergo a dramatic conformational change induced by binding of pro-apoptotic Bcl-2 activators (Bim, Puma and Noxa) [5]. Activators only harbor BH3 domains and are thus referred to as ‘BH3-only’ proteins.

Other Bcl-2 proteins block activators from interacting with Bak/Bax oligomers, and are termed ‘anti-apoptotic’ Bcl-2 proteins (Bcl2-A1, Bcl-2, Bcl-xL, Bcl-w and Mcl-1) [6–8]. Anti-apoptotic Bcl-2 proteins contain BH1-4 domains, which generate a hydrophobic pocket that tightly binds with BH3 domains in the BH3-only activators and in Bak/Bax [9, 10]. The BH3 motif of the anti-apoptotic Bcl-2 proteins is central to their function, and is the key to drug development strategies aimed at inhibiting anti-apoptotic Bcl-2 family members as a means to re-activate the intrinsic apoptotic pathway in cancer cells.

A final subclass of Bcl-2 proteins, the Bcl-2 sensitizers, is comprised of BH3-only proteins Bad, Bik, Hrk and Noxa that bind to BH1-4 domains of anti-apoptotic Bcl-2 proteins tightly, but do not bind to Bcl-2 effectors [7]. While sensitizers cannot activate Bak/Bax directly, their abundance can neutralize the inhibitor effects of the anti-apoptotic proteins, thus rendering cells more ‘sensitive’ to apoptotic stimuli. Thus, BH3-only proteins are pro-apoptotic forces that cooperate to enhance Bak/Bax-mediated MOMP. Importantly both BH3-only activators and sensitizers are rapidly upregulated in response to oxidative stress, DNA damage, endoplasmic reticulum stress or other insults that may provoke cell death through varying molecular mechanisms, including transcriptional regulation, subcellular localization and phosphorylation [11–14]. Because commitment to cell death occurs only when the complex interplay between pro- and anti-apoptotic Bcl-2 classes favors apoptosis (Figure 1B), the fluctuation in expression/localization/activation of BH3-only proteins in response to cellular cues liken the BH3-only proteins to a cellular rheostat, constantly adjusting and tuning the sensitivity of cells to apoptosis.

### Apoptosis is required in normal mammary gland development

The intrinsic cell death pathway is essential to several developmental phases of the mammary gland (Table 1). In response to hormonal stimuli during puberty, MECs within the terminal end buds (TEBs), the club-shaped structures at the distal mammary epithelial tips, proliferate and collectively invade the surrounding stroma. While differentiation of epithelial progenitors in the TEB populates the ducts with solid cords of mature luminal MECs, programmed cell death canalizes the ducts, resulting in lumen formation. Transgenic overexpression of anti-apoptotic Bcl-2 impaired TEB structure and disrupted ductal formation [15]. Similarly, genetic ablation of the BH3-only activator Bim delayed lumen formation due to reduced apoptosis in TEBs [16], demonstrating that Bcl-2 family proteins govern ductal lumen formation in the developing mammary gland during puberty.

**Table 1:** Transgenic misregulation of Bcl-2 family proteins in mouse models of mammary development and breast tumorigenesis

Class	BCL2 Factor	Genetically Engineered Model	Developmental Phenotype	Breast Cancer Phenotype	Ref.
Effector	Bak	n/a	n/a	n/a	n/a
	Bax	WAP-*Bax*	Impairs growth of alveolar MECs; premature apoptosis/involution	n/a	[1]
		MMTV-*Myc* X *Bax*+/−	n/a	Altered tumor multiplicity	[2]
		*Bax*−/−	Normal involution	Not highly tumorigenic	[3]
		*C3(1)-TAg X Bak*+/−	n/a	Reduces apoptosis at preneoplastic stage	[4]
Activator	Bim	*Bim*−/−	TEB/lumen filling	n/a	[10]
	Bid Puma	n/a	n/a	n/a	n/a
Sensitizer	Bad Bik Noxa	n/a	n/a	n/a	n/a
Anti-apoptotic	Bcl-2	WAP-*Bcl2*	Reduces apoptosis in TEBs, lumen filling during puberty; decreases cell death during involution	Accelerated MMTV-Myc-induced mammary tumorigenesis, carcinogen-induced breast tumor formation is delayed	[3, 5–7]
		WAP-*TAg*;WAP-*Bcl2*	na	Reduced tumor latency	[8]
	Bcl-xL	WAP-*Cre* X *Bcl-xL*^fl/fl^ MMTV-*Cre* X *Bcl-xL*^fl/fl^	Decreased apoptosis during involution	n/a	[9]
	A1 Bcl-w Mcl-1	n/a	n/a	n/a	n/a

n/a - not available.

1 Rucker, E.B., 3rd, et al., Forced involution of the functionally differentiated mammary gland by overexpression of the pro-apoptotic protein bax. Genesis, 2011. 49:24–35.

2 Jamerson, M.H., et al., Bax regulates c-Myc-induced mammary tumour apoptosis but not proliferation in MMTV-c-myc transgenic mice. Br J Cancer, 2004. 91:1372–9.

3 Schorr, K., et al., Gain of Bcl-2 is more potent than bax loss in regulating mammary epithelial cell survival in vivo. Cancer Research, 1999. 59:2541–5.

4 Shibata, M.A., et al., Haploid loss of bax leads to accelerated mammary tumor development in C3(1)/SV40-TAg transgenic mice: reduction in protective apoptotic response at the preneoplastic stage. EMBO J, 1999. 18:2692–701.

5 Jager, R., et al., Overexpression of Bcl-2 inhibits alveolar cell apoptosis during involution and accelerates c-myc-induced tumorigenesis of the mammary gland in transgenic mice. Oncogene, 1997. 15:1787–95.

6 Humphreys, R.C., et al., Apoptosis in the terminal endbud of the murine mammary gland: a mechanism of ductal morphogenesis. Development, 1996. 122:4013–22.

7 Murphy, K.L., et al., Bcl-2 expression delays mammary tumor development in dimethylbenz(a)anthracene-treated transgenic mice. Oncogene, 1999. 18:6597–604.

8 Furth, P.A., et al., Loss of anti-mitotic effects of Bcl-2 with retention of anti-apoptotic activity during tumor progression in a mouse model. Oncogene, 1999. 18:6589–96.

9 Walton, K.D., et al., Conditional deletion of the bcl-x gene from mouse mammary epithelium results in accelerated apoptosis during involution but does not compromise cell function during lactation. Mech Dev, 2001. 109:281–93.

10 Mailleux AA, Overholtzer M, Schmelzle T, Bouillet P, Strasser A, Brugge JS. BIM regulates apoptosis during mammary ductal morphogenesis, and its absence reveals alternative cell death mechanisms. Dev Cell, 2007. 12:221–234.

Once the ductal epithelium is established, other MECs throughout the breast, the alveolar progenitor cells, undergo hormonally-induced growth during the menstrual cycle (in humans) or estrus cycle (in rodents and other mammals). This expansion prepares the MEC population for rapid pregnancy-induced growth of the alveolar (milk-producing) epithelium. However, in the absence of pregnancy, the expanded alveolar progenitor population is removed by cell death. Thus, apoptosis within the breast occurs with each hormonal cycle throughout a woman's reproductive lifespan.

In the event of pregnancy massive expansion of the alveolar (milk-producing) epithelium occurs in preparation for nursing. The milk-producing cells are maintained while offspring continue to nurse. Once weaning occurs, the alveolar epithelium undergoes massive cell death in a tissue remodeling event termed post-lactational involution. During this short period of time (7–10 days in mice, 6–12 months in women), up to 80% of all MECs undergo cell death. Evidence of Bcl-2 family regulation of this event is abundant. High expression levels of the anti-apoptotic proteins Bcl-2 and Bcl-w during lactation are rapidly down-regulated during post-lactational involution [17], allowing for induction of cell death. Disruption of the Bcl-2 family balance through transgenic Bax overexpression results in cell death during lactation instead of during involution, resulting in premature involution [18]. Furthermore, genetic Bcl-xL ablation in the mammary epithelium dramatically increases cell death during post-partum involution [19]. Collectively, these studies demonstrate that Bcl-2 family proteins are key regulators of cell survival during lactation and are critical for inducing cell death of the milk producing epithelium once lactation ceases.

Less is known regarding the Bcl-2 family in lobular (menopausal) involution of the breast. However, increasing evidence suggests that the extent to which lobular involution occurs is directly proportional to a woman's risk of developing post-menopausal breast cancer. Nearly 70% of all breast cancers occur in post-menopausal women. It is therefore important to understand the molecular mechanisms contributing to lobular involution. Given the importance of Bcl-2 proteins in other stages of breast development, these observations support the exploration of Bcl-2 family proteins in lobular involution.

### Bcl-2 family in breast cancer formation and progression

Anti-apoptotic Bcl-2 proteins were first acknowledged as oncogenes in 1984 upon discovery of Bcl-2 dysregulation due to the chromosomal translocation t(14; 18) that was a driving factor in B-cell leukemia [20], changing the oncogene paradigm to include anti-apoptotic proteins. Evasion of cell death is now acknowledged as a hallmark of cancer, required to overcome the counterbalancing effects of cell death on enhanced cell proliferation [21]. As an illustration of this point, studies performed in three-dimensional acinar cultures of untransformed MECs demonstrated that oncogene-induced proliferation was insufficient to support lumen filling due to equally increased cell death [22, 23]. However, Bcl-2 and Bcl-xL over-expression in combination with oncogene-induced proliferation resulted in lumen filling, a morphological characteristic of ductal carcinoma *in situ* (DCIS), an early pre-malignant state [24]. These results demonstrated that pro-proliferative and anti-apoptotic signals cooperate in early MEC transformation and that anti-apoptotic Bcl-2 proteins contribute to this process.

Consistent with these findings, although transgenic overexpression of Bcl-2 alone did not generate mammary tumors, c-Myc induced [25] and SV40 Large T-antigen (TAg)-induced [26] mammary tumors developed at a remarkably faster rate in the context of Bcl-2 overexpression. Similarly, genetic Bax ablation accelerated TAg-induced mammary tumor progression [27]. In human breast cancers, up to 80% of *MYC*-amplified triple negative breast cancer (TNBC) cases harbor *MCL1* co-amplification [28]. Further, Bcl-2 expression frequently correlates with Estrogen Receptor (ER) expression levels in ER^+^ breast cancers [29]. Taken together, these studies suggest that anti-apoptotic Bcl-2 proteins may cooperate with pro-proliferative signals to support breast cancer initiation and progression.

### Bcl-2 family in therapeutic resistance

The goal of chemotherapies, therapeutic radiation and molecularly targeted drugs is effective tumor cell killing. However, this goal is often thwarted by increased anti-apoptotic and/or decreased pro-apoptotic Bcl-2 protein activity or expression. In the context of chemotherapy, clinical breast cancer specimens biopsied before and after a course of pre-operative neoadjuvant chemotherapy displayed increased Bcl-2 in residual post-treatment tumor cells [30]. Furthermore, metastatic human breast cancer cell lines with high Bcl-2 levels were less sensitive to taxanes [31] and Adriamycin [32]. *MCL1* amplification was more frequently observed in chemo-refractory TNBC samples as compared to the total TNBC sample population [28]. Together, these studies suggest that anti-apoptotic Bcl-2 family proteins interfere with chemotherapy-induced apoptosis in breast cancers.

Bcl-2 family proteins also mediate resistance to targeted breast cancer therapies. The anti-HER2 antibody, trastuzumab, is clinically approved for use in 20% of all breast cancers that exhibit *HER2* gene amplification. However, many *HER2*-amplified tumors display innate trastuzumab resistance, while others rapidly acquire trastuzumab resistance. Trastuzumab-resistant HER2^+^ breast cancer cell lines frequently upregulate Bcl-2 and decrease Bax as a means of enhancing cell survival [33].

Nearly 65% of breast cancers are ER^+^ and are thus treated with drugs that interfere with ER signaling, such as selective ER modulators (SERMS, e.g. tamoxifen), selective ER downregulators (SERDS, e.g., fulvestrant) or drugs that decrease circulating estrogen levels, like aromatase inhibitors (AIs, e.g., exemestane). Although these targeted therapies improve outcome for many patients with ER^+^ breast cancers, 15–20% relapse within 5 years of treatment withdrawal [34]. Tamoxifen treated ER^+^ breast cancers often increase Bcl-2 and Bcl-xL levels, decreasing acute tumor response to Tamoxifen and increasing long-term tamoxifen resistance [35]. Using estrogen deprivation of ER^+^ human breast cancer cell lines as a model of AI treatment, one study showed that cell survival under these conditions is supported by increased activity of Bcl-2 at the mitochondria [36]. Upon long-term estrogen deprivation, endoplasmic reticulum chaperone proteins sequester the pro-apoptotic Bcl-2 protein, Bik, preventing its translocation to the mitochondria, allowing Bcl-2 to inhibit Bak/Bax-mediated apoptosis. Thus, Bcl-2 protein dysregulation favoring expression or activity of anti-apoptotic family members is a driver of resistance to HER2 and ER-targeted therapies.

### Targeting Bcl-2/Bcl-xL/Bcl-w to enhance breast tumor cell killing

Based on observations that tumors require evasion of cell death, and that Bcl-2 family anti-apoptotic proteins are frequently overexpression or hyper-activated in cancers, it is likely that certain cancers maybe particularly sensitive to targeted inhibition of anti-apoptotic Bcl-2 proteins. This hypothesis is the basis for the relatively recent development of BH3-mimetics, a class of small molecule Bcl-2 family inhibitors that tightly bind the hydrophobic BH3-binding motif within anti-apoptotic Bcl-2 proteins, allowing for release of Bcl-2 activators from sequestration by Bcl-2 inhibitors, MOMP and caspase-dependent cell death [37]. Several BH3 mimetic have been developed, including the Bcl-2/Bcl-xL/Bcl-w inhibitor ABT-263 [38], which binds to Bcl-2, Bcl-xL and Bcl-w in a manner similar to the BH3-domain of the sensitizer BAD. Although ABT-263 has shown promising anti-cancer activity in chronic lymphocytic leukemia (CLL) as a single agent [39], ABT-263 caused on-target induction of apoptosis in platelets [40], limiting its clinical utility. The Bcl-2 selective inhibitor ABT-199 spares platelets, which require Bcl-xL signaling [41, 42]. Despite the suspension of initial clinical trials using ABT-199 in CLL patients due to fatal levels of tumor lysis, ABT-199 trails in CLL and other cancers are ongoing [43].

Although only limited information regarding BH3-mimetic in clinical breast cancers is available, pre-clinical models support the use of BH3-mimetics in combination with existing breast cancer treatment strategies. For example, Bcl-2 is overexpressed in about 85% of ER^+^ breast cancers, perhaps due to the fact that its promoter is directly bound and transactivated by ERα [44]. Patient-derived ER^+^ breast cancer xenografts treated with tamoxifen in combination with the Bcl-2/Bcl-xL/Bcl-w inhibitor ABT-737 [37], an analogue of ABT-263, displayed decreased tumor growth and increased tumor cell death [45]. Similar results were obtained using Tamoxifen in combination with ABT-199, the Bcl-2 selective inhibitor. However, BH3-mimetics alone failed to decrease tumor growth or induce apoptosis in this model. In models of TNBC, ABT-737 sensitized patient-derived basal-like breast cancer xenografts to the chemotherapeutic agent docetaxel [46] and increased cell death in irradiated TNBC in cell culture and *in vivo* [47]. GX15–070 (obatoclax), a pan Bcl-2 family inhibitor, cooperated with lapitinib (an EGFR/HER2 inhibitor) to induce cell death in HER2^+^ breast cancer cells treated with radiotherapies [48]. These studies suggest that targeting Bcl-2 family proteins in combination with current chemotherapies and targeted therapies may increase tumor cell killing and therefore improve the outcome of patients with breast cancer. Although no current clinical trials are studying the effects of anti-apoptotic Bcl-2 family member inhibition in breast cancers alone, initial Phase I studies in small cell lung cancers suggest that ABT-263 is safe and well tolerated in patients with solid tumors [49]. Additionally, Phase I studies are underway to determine the efficacy of ABT-263 with paclitaxel, gemcitabine and sorafinib, in solid tumors, which could include breast cancers [50–52].

### Targeting Mcl-1 in breast cancer

Although there is strong preliminary evidence supporting the roles of Bcl-2 and Bcl-xL in breast tumor formation and therapeutic resistance, high Bcl-2 protein levels in human breast cancer samples tend to correlate with a favorable prognosis [53]. Furthermore, according The Cancer Genome Atlas (TCGA) genetic amplification of *Bcl2* and *Bcl2l1* (encoding Bcl-xL) occurs in less than 3% of luminal B and TNBC cases [54], implying that BH3-mimetic targeting of Bcl-2 and Bcl-xL may not be beneficial in most breast cancer subtypes. However, targeting of other anti-apoptotic Bcl-2 family members, like Mcl-1, may provide an alternative approach.

**Figure 2:**
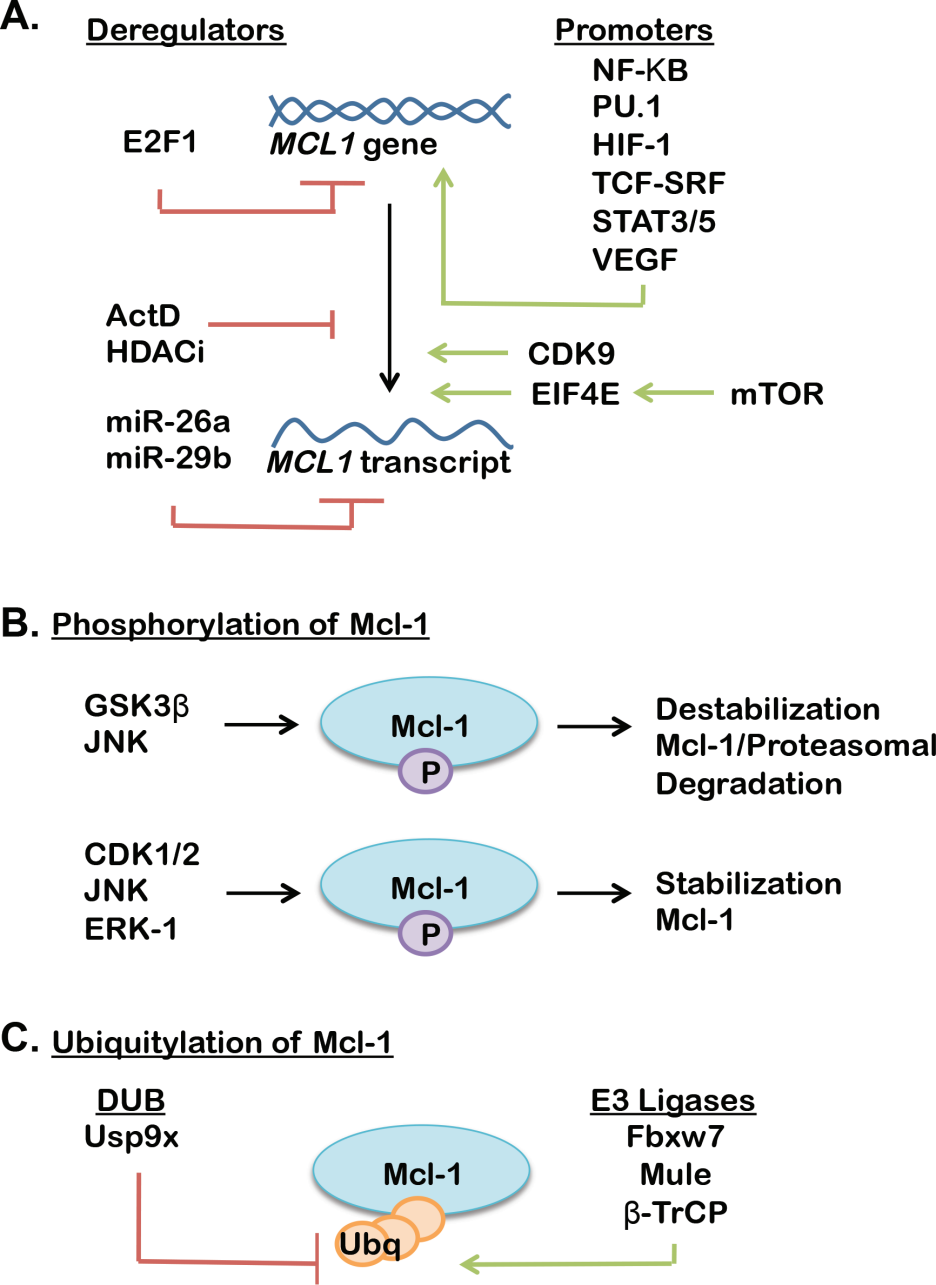
**(A)** Transcriptional, translational and post translational mechanisms of Mcl-1 regulation (promoters support Mcl-1 expression, while deregulators decrease Mcl-1 expression). **(B–C)** A complex interplay of phosphatases (B), ubiquitinases and deubiquitinases (DUBs) (C) regulate Mcl-1 stability through activation or suppression of ubiquitin-mediated proteasomal degradation of Mcl-1 (further reviewed in [55, 56]).

Currently, little is known regarding the contribution of Mcl-1 to breast cancer formation and therapeutic response. However, unlike other Bcl-2 family members, Mcl-1 has a short half-life and is highly regulated at the transcriptional, post-transcriptional, translational and post-translational levels [55, 56] by various oncogenic signaling pathways (Figure 2), including the mitogen activated protein kinase (MAPK) pathway [57], the mTOR pathway [58] and the phosphatidylinositol-3 kinase (PI3K) pathway [59]. These oncogenic signaling pathways rapidly increase Mcl-1 to promote cell survival, suggesting that Mcl-1 may be an important mediator of apoptotic escape and therapeutic resistance in many cancers, including breast cancers. This hypothesis is supported by the fact that the dual PI3K/mTOR inhibitor NVP-BEZ235 was found to decrease Mcl-1 levels in ovarian carcinomas cells [60]. Additionally, Mcl-1 expression was inhibited by targeting of the mTOR pathway using the rapalouge RAD001/everolimus, promoting increased tumor cell killing in *in vivo* models of colon cancer [61].

**Figure 3:**
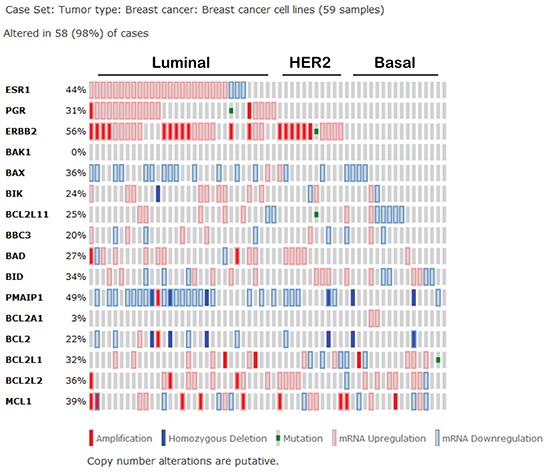
Expression signature of Bcl-2 family proteins in human breast cancer cell lines according to the cancer cell line encyclopedia as curated by the cancer genome atlas (BCL2L11 = BIM, BBC3 = PUMA, PMAIP1 = NOXA, BCL2L1 = Bcl-xL, BCL2L2 = Bcl-w).

Specifically in the context of breast cancer, Mcl-1 has genetic or mRNA upregulation in 16/58 (28%) of human breast cancer cell lines as curated by the CCLE (Figure 3), while Bcl-2 is only aberrantly upregulated in 2/58 cases (3%) [54]. Additionally, expression of Mcl-1 in human breast cancer samples correlates with high tumor grade and a dramatic decrease in patient survival regardless of subtype [62]. *MCL1* is also genetically amplified in 9% of luminal B breast cancers [54] and 54% of TNBCs after treatment with neoadjuvant chemotherapies [28], suggesting that Mcl-1 may represent a more desirable target over Bcl-2/Bcl-xL in some breast cancers. Indirect evidence that targeting of Mcl-1 in combination with lapitinib could sensitize breast cancer cells to radiotherapies was gained using the pan-BCL2 inhibitor obatoclax, a BH3-mimetic that displaces BIM from all anti-apoptotic BCL2 family members. These preliminary studies suggest that Mcl-1 may support the escape of breast cancer cells from therapy-induced cell death (Figure 1C), suggesting that targeting Mcl-1 may be beneficial to improving the outcome of breast cancer patients, particularly those with Mcl-1 amplification [63].

The importance of targeting Mcl-1 in breast cancers is further supported by extensive studies conducted on anti-apoptotic Bcl-2 family proteins in other cancers. These studies suggest that high Mcl-1 expression or Mcl-1 upregulation confer resistance to the Bcl-2/Bcl-xL/Bcl-w inhibitor ABT-263 in leukemias, lymphomas, melanomas and lung cancers [64–69]. These findings highlight the potential compensatory nature of anti-apoptotic Bcl-2 family members and suggest that the maximum tumor cell killing may only be achieved under conditions where Bcl-2, Bcl-xL, Bcl-w and Mcl-1 are inhibited (Figure 1D). To overcome this limitation, recent efforts have focused on isolating and developing an Mcl-1 specific inhibitor, as reviewed by Belmar J and Fesik 2014 [70]. Unfortunately, these efforts have been met with limited success due to: (1) conflicting reports on the efficacy of these drugs to inhibit Mcl-1 in various cancer cell lines, (2) low affinity of these drugs for Mcl-1 and (3) lack of *in vivo* data supporting the efficacy of these inhibitors. However, the discovery of an Mcl-1-specific inhibitor is still a relatively new field and further efforts may identify a potent Mcl-1-speicifc inhibitor with immense clinical/translational value.

Although preliminary studies in breast cancers demonstrate that inhibiting Bcl-2 or Bcl-2/Bcl-xL/Bcl-w in combination with targeted therapies is sufficient to dramatically increase tumor cell killing and survival, this might not be the case for all breast cancers, particularly those with *MCL1-*amplification. Instead, Mcl-1 alone, or in combination with other anti-apoptotic Bcl-2 family members, may be an essential driver of tumor progression and mediator of therapeutic resistance in breast cancers.

### Future directions

Mcl-1 remains understudied in breast cancers as compared to other Bcl-2 family anti-apoptotic proteins. Regardless, it is clear that anti-apoptotic Bcl-2 family members influence the formation, progression and therapeutic response of breast cancers. Recent efforts in developing an Mcl-1 specific inhibitor have been reported [71, 72], although their efficacy in breast cancers and pre-clinical testing remains on the horizon. Currently, inhibition of the numerous pathways that regulate Mcl-1 expression and stability are viable strategies to block the oncogenic effects of Mcl-1 (Figure 2). Importantly, inhibitors for many of these pathways are approved for clinical use in many cancers, including inhibitors of MAPK, mTOR and PI3K pathways. These inhibitors may provide a feasible and rapidly translatable approach to limiting Mcl-1 activity in breast cancers. A potential disadvantage to this approach is deciphering which pathway is the most reasonable for achieving targeted Mcl-1 inhibition for each patient. Further, since multiple signaling pathways converge on Mcl-1 numerous mechanisms exist to re-establish Mcl-1 signaling, suggesting that many avenues exist to promote therapeutic escape. Thus, the observations highlighted in this review support a continued effort to design Mcl-1-specific inhibitors, while investigation into the role of Mcl-1 in mammary gland development and breast cancer will be essential to maximize the clinical value of emerging Mcl-1 inhibitors.
